# Decreased acylated and total ghrelin levels in bipolar disorder patients recovering from a manic episode

**DOI:** 10.1186/s12888-022-03842-1

**Published:** 2022-03-21

**Authors:** Karim Abdel Aziz, Fadwa Al-Mugaddam, Subi Sugathan, Prashanth Saseedharan, Tarek Jouini, Mohamed Elhassan Elamin, Hamdy Moselhy, Dina Aly El-Gabry, Danilo Arnone, Sherif M. Karam

**Affiliations:** 1 Department of Psychiatry and Behavioural Sciences, College of Medicine and Health Sciences, United Arab Emirates University, Al Ain, United Arab Emirates; 2 Department of Anatomy, College of Medicine and Health Sciences, United Arab Emirates University, Al Ain, United Arab Emirates; 3 Dept of Chemical Engineering and Biotechnology, University of Sherbrooke, Sherbrooke, QC, Canada; 4 Behavioural Science Institute, Al-Ain Hospital, Al-Ain, United Arab Emirates; 5 Drogheda Department of Psychiatry, Drogheda, County Louth, Ireland; 6 American Center for Psychiatry and Neurology (ACPN), Dubai, United Arab Emirates; 7 Okasha Institute of Psychiatry, Neuropsychiatry Department, Ain Shams University, Cairo, Egypt; 8 Institute of Psychiatry, Psychology and Neuroscience, Centre for Affective Disorders, Kings’ College London, London, UK

**Keywords:** Bipolar disorder, Mania, Euthymia, Total ghrelin, Acylated ghrelin, Des-acylated ghrelin

## Abstract

**Background:**

To date, only few studies have investigated ghrelin levels in bipolar disorders, and all have exclusively measured acylated ghrelin, with none investigating total ghrelin (acylated and des-acylated). We aimed to investigate peripheral levels of acylated and total ghrelin in subjects experiencing a manic episode of bipolar disorder.

**Methods:**

Peripheral levels of acylated and total ghrelin were measured in hospitalised medicated individuals recovering from a manic episode. Enzyme-linked immunosorbent assays (ELISA) were used to measure ghrelin levels in patients and compared with healthy controls. The relationship between ghrelin levels in bipolar disorder, self-reported hunger measures, demographic and clinical parameters was investigated with correlational analyses.

**Results:**

Twenty-four subjects (15 males, 9 females) recovering from mania and 27 matched healthy controls (13 males, 14 females) were recruited for the study. Mean values of both acylated (187 vs.520 pg/mL) and total ghrelin (396 vs. 648 pg/mL) were significantly reduced in bipolar disorder (*p* = 0.001). Ghrelin levels correlated positively with markers of illness severity and negatively with prescribed mood stabilizers, second-generation antipsychotics, weight and body mass index.

**Conclusion:**

Peripheral measurements of acylated and total ghrelin were both reduced in bipolar disorder patients compared to healthy controls. Whilst illness severity promotes higher ghrelin levels, pharmacological treatment and weight gain exercise the opposite effect.

**Supplementary Information:**

The online version contains supplementary material available at 10.1186/s12888-022-03842-1.

## Introduction

Bipolar disorders are mood disorders characterized by the presence of episodes of depression and mania with a lifetime prevalence of 1 to 3% [1]. Globally bipolar disorders are the sixth cause of disability affecting about 45 million people in 2016 [2, 3].

There is great interest in improving our understanding of the biology of bipolar disorders with the hope that this might improve therapeutic targets and shed some light on the higher-than-expected level of physical morbidity and mortality, often complicated by metabolic abnormalities and high body weight [4]. Research suggests that ghrelin, a peripheral hormone known to regulate food intake, may be a relevant player in bipolar disorders.

Ghrelin is a 28-amino acid peptide hormone with multiple biological functions. It triggers the release of growth hormone (GH) by acting on the GH secretagogue receptor 1a (GHSR) [5]. It also exercises orexigenic effects (hunger stimulation promoting food intake), modulates glucose homeostasis, regulates lipid metabolism and enhances gastrointestinal motility and regeneration [6–9]. In the central nervous system ghrelin is involved in cognition, mood, reward and sleep [10–12] through the modulation of GHSRs in the hypothalamic–pituitary axis, hippocampus, substantia nigra, ventral tegmental area, raphe nuclei, Edinger–Westphal nucleus and pyriform cortex [13]. Hypothalamic-limbic connections where GHSR are expressed may explain the possible link between ghrelin and alterations in mood states [14]. Additionally, ghrelin interacts with brain reward pathways to increase food intake and enhance food reward [15]. There is evidence for an overlap of neurobiological mechanisms regulating both food and other reward pathways [16] which might be linked with impulsivity and risk taking. This suggests that ghrelin may have a role in increasing motivation and goal-directed behaviour, which are markedly increased in the manic phase of bipolar disorders and are part of their diagnostic criteria [17].

Whilst a small proportion of ghrelin is produced in the hypothalamus and pituitary gland, 60–70% of the circulating ghrelin originates from the gastric enteroendocrine cells with a small contribution from the small intestine, pancreas, lungs, kidneys, adrenal cortex, bones, testis and placenta [6, 18].

The two peripheral isoforms of ghrelin in humans include acylated and des-acylated ghrelin. Although acylated ghrelin accounts for less than 10% of the total circulating ghrelin it is historically considered the active peptide. The des-acylated isoform of ghrelin, which constitutes the majority of the circulating isoform [19], has more recently been shown to play a significant role in the metabolism of lipids, muscle, bone and endothelial cells [13, 20–22]. In addition, des-acylated ghrelin has been shown to be a functional antagonist of acylated ghrelin, and to exercise independent hormonal effects on a subset of cells of the arcuate nucleus in a GHSR-independent manner, influencing glucose and lipid metabolism with effects on cardiovascular, neurological, gastrointestinal, gonadal function [23, 24].

To date, studies of circulating peripheral levels of ghrelin in bipolar disorder suggest both elevated [25, 26] and reduced activity [27–29]. This can be related to different mood states, with some of the studies exploring either depressed or euthymic states, and some using mixed samples with different mood states. In one study, the ghrelin levels in patients during mania did not significantly differ compared to controls at baseline although the levels increased following response to treatment and improvement [26]. In addition, studies of ghrelin in bipolar disorders have exclusively investigated acylated ghrelin, with none evaluating total ghrelin (acylated and des-acylated ghrelin) considering that des-acyl ghrelin is equivalent to about 90% of the circulating hormone [9]. Furthermore, there is accumulating evidence that des-acylated ghrelin exerts a significant effect and is not inactive as initially thought.

Therefore, the aim of this study was to investigate peripheral levels of acylated and total ghrelin in bipolar disorder patients hospitalized for a manic episode and in relation to hunger measures, demographic and clinical variables. Based on previous work, we hypothesized that levels of total and acylated ghrelin levels will be higher in medicated bipolar subjects experiencing a manic episode than healthy controls although negatively influenced by body mass index.

## Methods

### Participants

In this cross-sectional study, cases were recruited from the psychiatry inpatient department at Al-Ain Hospital in the Emirate of Abu Dhabi, United Arab Emirates over a 3-year period. We included male and female subjects, aged 18–65 years, diagnosed with a manic episode of bipolar disorder at the time of admission according to the Diagnostic and Statistical Manual version 5 [17]. Prospective participants were recruited when able to consent to the study at week 4 after admission when symptoms were milder. Severity was established with the Young Mania Rating Scale (YMRS) [30]. A minimum score of 12 indicated the presence of manic symptoms. Co-morbid axis I or axis II psychopathology and history of substance misuse disorders were excluded. Healthy controls were matched according to demographic variables and were recruited from non-consanguineous relatives of patients visiting the hospital (e.g., spouse, spouse’s sibling), and from employees and students based at Al-Ain Hospital. History of any psychiatric disorder was an exclusion for healthy controls. For each participant, data collected included age, sex, weight (kg), height (m), BMI (kg/m^2^), family history of psychiatric disorders, past medical history, acylated and total ghrelin levels (pg/mL). Subjective sense of hunger was measured with the Visual Analogue Hunger Scale with scores varying from 1 meaning ‘not hungry at all’ to 10 being ‘very hungry’ [31].

In addition, for bipolar subjects, data collected included prescribed medication and clinical variables (index episode with bipolar disorder subtypes including duration in months, length of illness in years, number of hospitalizations and history of psychotic features).

### Measures of ghrelin levels

Blood samples (5 ml) were collected between 9 am-12 pm and drawn into anticoagulant-free serum tubes. Participants were non-fasting before venepuncture. Pefabloc (1 mg/ml) was added to inhibit proteases and the blood samples were allowed to clot at room temperature for 30 min. The samples were then centrifuged at 2500 x g for 15 min at 4 °C. Serum samples were collected and acidified with a final concentration of 0.05 N hydrochloric acid and stored at − 80 °C. Total and acylated ghrelin levels in serum were determined using 96-well plate total and active human ELISA kits (Merck Millipore, Missouri, USA), as per manufacturer’s instructions. Briefly, sandwich ELISA was performed by capturing acylated and total ghrelin molecules in 20 μl serum samples with specific antibodies. Then the ghrelin-antibody complex was immobilized to the bottom of the ELISA plate wells with the help of pre-treated anchor antibodies. The wells were incubated with biotinylated secondary antibodies, followed by streptavidin conjugated with horseradish peroxidase. Finally, the samples were treated with 3,3′,5,5′-tetramethylbenzidine substrate and the peroxidase activity was measured spectrophotometrically at 450 and 590 nm. Concentrations of both total and acylated ghrelin were calculated by using standard curves of their known concentrations.

### Statistical analyses

Data were recorded and analysed using the statistical package of social sciences IBM SPSS Statistics for Windows, version 26.0 (IBM Corp., Armonk, New York, USA). Results were tabulated, grouped and statistically analysed by using conventional approaches for parametric, non-parametric and non-numerical data. Chi-square test was used to compare categorical variables and independent-sample student t-tests to compare means. Univariate analysis of covariance (ANCOVA) within the General Linear Model was used to compare serum total and acylated ghrelin. The model was chosen as it takes into consideration potential unmatched covariates of interest. Pearson correlation coefficient (r) and point-biserial correlation test (r_pb_) for categorical data were used to study the direction and power of relationships. The analyses were Bonferroni corrected for multiple comparisons. Statistical significance was set at *p* ≤ 0.05.

## Results

### Demographic and clinical characteristics

Fifty-one subjects participated in the study, 24 bipolar disorder patients and 27 matched healthy subjects (see Table 1 for demographic and clinical characteristics). BMI and number of physical co-morbidities were significantly higher in patients compared to controls (29.8 vs. 26.4 and 9 vs. 3 respectively). At the time of the assessment, 19 patients had a history of psychotic symptoms, and all were medicated. The mean YMRS score suggested that patients were mildly manic or euthymic at the time of recruitment. Self-reported hunger scores did not differ between cases and controls. Medication classes included a range of different compounds whilst ghrelin levels varied within each pharmacological group (see Table 1).

**Table 1:** Demographic and clinical parameters in bipolar disorder patients and healthy controls

	Bipolar disorder (*N* = 24)	Healthy Controls (*N* = 27)	Test	*p* value
*Age in Yrs. (Mean & SD)*	31.92 (9.789)	34.41 (9.316)	t = −0.931	0.357
*M:F ratio*	15:9	13:14	χ2 = 1.057	0.304
*YMRS score (Mean & SD)*	8.58 (5.656)	–	–	–
*Illness duration in years (Mean & SD)*	4.42 (4.596)	–	–	–
*Episode duration in months (Mean & SD)*	2.25 (0.442)	–	–	–
*N. total Episodes (Mean & SD)*	2.04 (2.236)	–	–	–
*N. Manic Episodes (Mean & SD)*	1.67 (2.334)	–	–	–
*N. Depressive Episodes (Mean & SD)*	0.33 (0.702)	–	–	–
*N. of Mixed Episodes (Mean & SD)*	0.25 (0.532)	–	–	–
*N. of Hospitalizations (Mean & SD)*	2.67 (4.949)	–	–	–
*Past Medical History (N)*	9	3	χ2 = 4.917	0.027 *
*Weight in kgs (Mean & SD)*	81.47 (16.627)	74.43 (16.592)	t = 1.510	0.138
*Height in metres (Mean & SD)*	1.65 (0.095)	1.67 (0.084)	t = −0.559	0.579
*BMI-kg/m*^*2*^ *(Mean & SD)*	29.77 (6.648)	26.42 (4.604)	t = 2.109	0.040 *
*FH Psychiatric Disorder (N)*	3	2	χ2 = 0.373	0.542
*Hunger Score (Mean & SD)*	2.92 (1.998)	3.26 (1.457)	t = −0.705	0.484
*Acylated Ghrelin in pg/mL (Mean & SE)*	187 (69.46)	520 (60.79)	*F* = 12.211	0.001*
*Total Ghrelin in pg/mL (Mean & SE)*	396 (52.41)	648 (48.06)	*F* = 11.697	0.001*
*Pharmacological compounds*	***Total Ghrelin pg/mL (Mean & SD)***	***Acylated Ghrelin pg/mL (Mean & SD)***	*–*	*–*
*Mood Stabilizers (N = 20)*	407 (162.77)	172 (150.70)	–	–
*First Generation Antipsychotics (N = 3)*	485 (202.76)	197 (223.57)	–	
*Second Generation Antipsychotics (N = 16)*	395 (162.43)	216 (197.57)	–	–
*Third Generation Antipsychotics (N = 8)*	390 (144.88)	132 (47.38)	–	–
*Benzodiazepines (N = 13)*	408 (141.06)	255 (194.75)	–	–
*Hypnotics (N = 10)*	*347 (145.61)*	*237 (207.44)*	*–*	*–*
*Antidiabetics (N = 4)*	*364 (114.16)*	*318 (196.02)*	*–*	*–*
*Others (N = 7)*	*460 (185.40)*	*172 (162.29)*	*–*	*–*

N = number; *p statistically significant; SD=Standard Deviation; SE = Standard Error; Yrs = years; M: F = Male: Female; kg = kilograms; BMI = Body Mass Index; FH = Family History; YMRS = Young Mania Rating Scale; FGA = First Generation Antipsychotics; SGAs = Second Generation Antipsychotics; TGA = Third Generation Antipsychotics; pg/mL = picogram/ milliliter; χ2 = Chi-square; t = student t-test; *F* = ANCOVA

### Ghrelin level in bipolar subjects in comparison with healthy controls

**Fig. 1:**
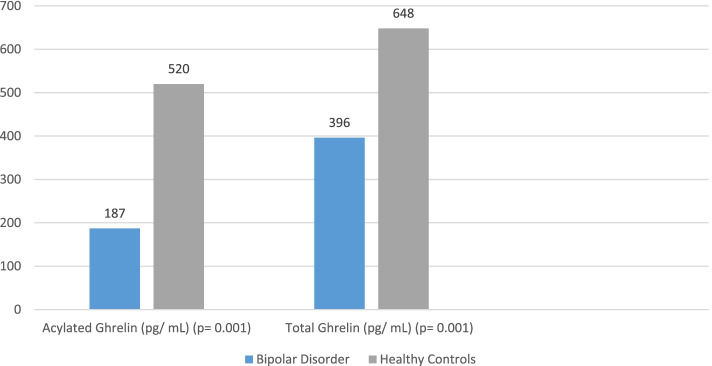
Acylated and total ghrelin levels in bipolar disorder in comparison with healthy controls

The analysis of covariance which incorporated BMI and past medical history as covariates of interest indicated that mean levels of acylated and total ghrelin were significantly lower in bipolar patients vs. healthy controls (both *p* = 0.001) (see Table 1 and Fig. 1 for details).

### Relationship between total and acylated ghrelin levels with demographic, clinical parameters and pharmacological treatment in bipolar disorder

The level of acylated ghrelin was higher in patients who experienced more manic episodes (*r* = 0.674, *p* = 0.001) and hospitalizations (*r* = 0.462, *p* = 0.035) and lower with increasing weight (*r* = − 0.339, *p* = 0.018) and BMI (*r* = − 0.326, *p* = 0.024). Levels of acylated and total ghrelin were higher in females (*r*_*pb*_ = 0.331, *p* = 0.019 for total ghrelin; *r*_*pb*_ = 0.305, *p* = 0.035 for acylated ghrelin) and lower in patients receiving mood stabilizers (*r*_*pb*_ = − 0.368, *p* = 0.008 for total ghrelin; *r*_*pb*_ = − 0.425, *p* = 0.003 for acylated ghrelin) or second-generation antipsychotics (*r*_*pb*_ = − 0.337, *p* = 0.017 for total ghrelin; *r*_*pb*_ = − 0.288, *p* = 0.047 for acylated ghrelin). The level of total ghrelin was lower in patients receiving hypnotics (*r*_*pb*_ = − 0.327, *p* = 0.021). There were no significant effects of subjective hunger measurement and of YMRS scores or of any other variables (See supplementary Table 2).

## Discussion

To the best of our knowledge, this study is the first to investigate levels of both total and acylated ghrelin in patients with bipolar disorder. The work also investigated demographic and clinical parameters associated with changes in ghrelin levels. Differently than expected, results showed that patients who experienced a recent manic episode had significantly lower levels of both acylated and total ghrelin compared to healthy controls after controlling for possible confounders. Hence, we found that overall measures of acylated and total ghrelin were synergistic.

Interestingly, correlations suggested that the level of acylated ghrelin was higher in patients who experienced more manic episodes and hospitalizations and lower with increasing weight and BMI. Levels of acylated ghrelin were higher in females than males. Overall, the data support the notion that acylated peripheral levels might be negatively affected by BMI and weight, particularly in men, whilst higher levels of ghrelin might be a proxy for more severe forms of illness. We also found that pharmacotherapy (mood stabilizers or a second-generation antipsychotics) correlated negatively with both acylated and total ghrelin levels, a direction of the effect opposite to parameters suggesting a more severe index episode.

In relation to our main finding of reduced acylated and total ghrelin levels, Kalenderoğlu and colleagues reported lower levels of ghrelin in euthymic patients with bipolar disorder receiving medication for at least 3 months compared to healthy controls [28]. This is somewhat similar to the data presented in this work which included medicated patients recruited within 4 weeks of recovery from a recent manic episode. Tunçel and colleagues did not find that acylated ghrelin levels varied in patients experiencing mania vs. healthy controls [26]. In another study, acylated ghrelin levels significantly increased in manic patients after treatment and symptoms amelioration. The authors attributed these findings to the possible effect of olanzapine in inhibiting post-prandial reduction in ghrelin levels [32] corroborated by further evidence that olanzapine might enhance post-prandial activation of GHSRs [33].

While our study was cross-sectional and did not specifically investigate the effect of treatment on ghrelin levels, we did find significant negative correlations between the intake of mood stabilizers, atypical antipsychotics with acylated and total ghrelin levels. This finding is consistent with studies suggesting that mood stabilizers (particularly valproate in epileptic patients) and atypical antipsychotics tend to reduce ghrelin levels [34, 35].

There is also evidence that bipolar disorder patients receiving lithium monotherapy display significantly lower levels of ghrelin compared to controls [27]. Similarly, bipolar patients with mania receiving electroconvulsive therapy were found to have significantly lower ghrelin levels post-treatment compared to pre-treatment [36]. Collectively, these findings suggest the possibility that reduced ghrelin levels in bipolar disorder patients might be associated with pharmacological treatment, although some treatment types like olanzapine might exercise the opposite effect.

Güler and colleagues reported increased ghrelin levels in bipolar disorder patients experiencing a depressive episode and in remission compared to healthy controls [25]. These differences were attributed to some of the symptoms such as disturbance in food intake and sleep-wake dysregulation. Rosso and colleagues found significantly lower levels of ghrelin in patients with bipolar depression compared to controls and suggested that this might play a role in the pathogenesis of bipolar disorder representing a state-marker in the absence of significant medication effects [29]. The significant positive correlation between acylated ghrelin and numbers of hospitalization might at face value support the possibility of an association between increasing levels of ghrelin and chronicity as suggested by staging illness models proposing that a high number of recurrent episodes reflects late-stage bipolar disorder [37].

Our study found a significant negative correlation between acylated ghrelin levels with body weight and BMI. Previous studies in bipolar disorders showed mixed results regarding this aspect of ghrelin functions with one study demonstrating no correlation between BMI and ghrelin levels [36] and two others showing that BMI negatively correlated with ghrelin levels, in agreement with our findings [29, 38]. Ghrelin levels tend to rise in states of weight loss in the context of caloric restriction, cachexia, anorexia nervosa and bulimia nervosa and decrease in states of overfeeding. This ghrelin response is reported not to be dependent from food intake per se as evidenced by rising ghrelin levels in weight loss through exercise in the absence of food restriction. Interestingly we did not find any relationship between ghrelin isoforms and self-reported measures of hunger. Ghrelin is therefore very likely to play a significant wider role in energy homeostasis [39] and together with other metabolic parameters [29] might be of interest in bipolar disorders especially in view of the reported physical morbidity and mortality [4].

Our study showed a significant correlation between sex and total and acylated ghrelin with higher levels in women. Previous studies exploring sex differences and ghrelin levels in bipolar disorders are contradictory. Kurt and colleagues found no correlation between ghrelin level and sex attribution [36] and Kalenderoğlu and colleagues found no significant sex effects in ghrelin levels of bipolar disorder patients [28]. On the other hand, Rosso and colleagues found a significant correlation between sex and ghrelin levels [29] whilst Kim and colleagues reported higher acylated ghrelin levels in men [38]. Makovey and colleagues noted that sex differences tended to vary according to the BMI range of the groups under investigation with significantly higher ghrelin levels reported in women in the normal BMI range, and no sex effects in case of obesity [40]. The latter findings suggest higher levels of ghrelin in women with a plateau for measures of adiposity beyond which sex differences become less relevant. The participants of this study were largely not in the obese range which might justify our findings in line with previous research.

Limitations of this study include, in line with similar work, the relatively small sample size. This reflects the genuine difficulty in recruiting mood disorder patients [41]. In addition, patients admitted in the manic phase were recruited when symptoms were sufficiently mild to allow informed consent to take place. It follows that there are limitations in the generalizability of our findings in relation to severity and other phases of the illness (e.g., depressive, mixed phase). In addition, the very cross-sectional nature of the study does not allow inferences related to causality. A longitudinal study might be better equipped to address this issue. We also included patients receiving heterogeneous medications that could affect metabolic parameters including appetite. Although we did not find any effect of self-reported satiety in our analyses, it would be preferable to investigate medication free patients, although it may be difficult to justify delaying treatment from an ethical perspective. Furthermore the 4-week gap between patients being admitted and being recruited into the study created a time lag which prevented the study from investigating mania in the very acute phase. Altered levels of catecholamines and stress-related hormones such as cortisol, shown to be associated with bipolar disorders [42–44], were not evaluated in this study and could have contributed to the measured suppressed levels of ghrelin. Hence it is important to consider controlling for these factors in future studies to establish their relationship with ghrelin. Also, this study did not include measurements of levels of leptin. This information could have contributed to further understand the relationship between ghrelin and leptin in bipolar disorders. There is however evidence that leptin levels are not altered in bipolar disorder across the mood spectrum compared to healthy controls [24]. The indirect measurement of the des-acylated isoform from the total ghrelin in a similar or lower range than acylated ghrelin levels, suggests that the stabilisation of the patient samples was optimal and that blood samples were adequately processed.

## Conclusions

This study indicates that measures of acylated and total ghrelin were synergistic. Both acylated and total ghrelin levels were decreased in medicated patients with bipolar disorder recovering from a manic episode. There is no evidence from this study that self-reported hunger measures differed in bipolar disorder or impacted on ghrelin levels. In addition, lower levels of ghrelin tended to be associated with higher weight and BMI. The level of ghrelin tended to increase with illness severity whereas prescribed medication exercised somewhat the opposite effect. Enthusing evidence emerging from neuroimaging studies might offer interesting biological models for early detection and identification of potential neurobiological targets in mood disorders which could include ghrelin [45, 46]. Further preclinical and clinical investigations are necessary to better understand whether ghrelin plays a role in the pathophysiology, progression, physical morbidity and mortality, response and remission of bipolar disorders.

## Supplementary Information

**Additional file 1.**

## Data Availability

The datasets generated and/or analysed during the current study are not publicly available due to restrictions of data sharing but are available from the corresponding author on reasonable request.
