# Supplementary material for: Decreased acylated and total ghrelin levels in bipolar disorder patients recovering from a manic episode
Source: BMC Psychiatry. 2022 Mar 21;22:209. doi: 10.1186/s12888-022-03842-1 (PMC8935687; doi:10.1186/s12888-022-03842-1)
Supplement: Supplementary file 1 — Additional file 1. [file 12888_2022_3842_MOESM1_ESM.docx]

|  | **Total Ghrelin level**  **(*pg/mL)*** | | **Acylated Ghrelin level**  **(*pg/mL)*** | |
| --- | --- | --- | --- | --- |
|  | ***r*** | ***p* value** | ***r*** | ***p* value** |
| ***Age in years*** | 0.043 | 0.765 | -0.031 | 0.834 |
| ***Weight (kg)*** | -0.245 | 0.086 | -0.339 | 0.018* |
| ***Height (m)*** | -0.135 | 0.349 | -0.130 | 0.380 |
| ***BMI (kg/m^2^)*** | -0.200 | 0.164 | -0.326 | 0.024* |
| ***Hunger Score*** | 0.019 | 0.897 | 0.152 | 0.304 |
| ***Number of Total Episodes*** | -0.390 | 0.065 | 0.415 | 0.061 |
| ***Duration of Episode*** | 0.190 | 0.386 | 0.263 | 0.250 |
| ***Duration of Illness*** | -0.041 | 0.852 | 0.024 | 0.916 |
| ***Number of Manic Episodes*** | -0.341 | 0.111 | 0.674 | 0.001* |
| ***Number of Depressive Episodes*** | -0.235 | 0.281 | -0.268 | 0.240 |
| ***Number of Mixed Episodes*** | 0.182 | 0.407 | -0.241 | 0.293 |
| ***Number of Hospitalizations*** | -0.314 | 0.144 | 0.462 | 0.035* |
| ***YMRS total score*** | 0.103 | 0.641 | 0.159 | 0.490 |
| ***Elevated mood*** | 0.034 | 0.878 | 0.145 | 0.530 |
| ***Increased motor activity/ energy*** | 0.031 | 0.889 | 0.289 | 0.204 |
| ***Sexual interest*** | -0.175 | 0.425 | 0.358 | 0.111 |
| ***Sleep*** | -0.091 | 0.681 | 0.073 | 0.754 |
| ***Irritability*** | -0.156 | 0.478 | -0.291 | 0.200 |
| ***Speech (rate and amount)*** | 0.121 | 0.584 | 0.022 | 0.926 |
| ***Language/ thought disorder*** | -0.122 | 0.579 | 0.133 | 0.565 |
| ***Content*** | 0.018 | 0.937 | 0.111 | 0.632 |
| ***Disruptive/aggressive behavior*** | 0.088 | 0.691 | 0.081 | 0.726 |
| ***Appearance*** | 0.245 | 0.261 | 0.204 | 0.374 |
| ***Insight*** | 0.355 | 0.097 | 0.054 | 0.817 |
|  | ***r_pb_*** | ***p value*** | ***r_pb_*** | ***p* value** |
| ***Female Sex*** | 0.331 | 0.019* | 0.305 | 0.035* |
| ***+ve Past Medical History*** | 0.060 | 0.678 | -0.061 | 0.679 |
| ***History of Psychotic Symptoms*** | -0.010 | 0.963 | -0.238 | 0.262 |
| ***Mood Stabilizers*** | -0.368 | 0.008* | -0.425 | 0.003* |
| ***Second Generation Antipsychotics*** | -0.337 | 0.017* | -0.288^*^ | 0.047* |
| ***Third Generation Antipsychotics*** | -0.234 | 0.102 | -0.284 | 0.050 |
| ***First Generation Antipsychotics*** | -0.044 | 0.760 | -0.130 | 0.377 |
| ***Benzodiazepines*** | -0.278 | 0.051 | -0.185 | 0.209 |
| ***Hypnotics*** | -0.327 | 0.021* | -0.188 | 0.201 |
| ***Antidiabetics*** | -0.187 | 0.193 | -0.049 | 0.743 |
| ***Others*** | -0.110 | 0.447 | -0.238 | 0.103 |

**Supplementary Table:** Relationship between total, acylated ghrelin levels and demographic and clinical parameters of patients with bipolar disorder. *r*= Pearson correlation coefficient; *r_pb_* = point biserial correlation coefficient; * *p* statistically significant; kg= kilograms; m= metres; BMI= Body Mass Index; YMRS= Young Mania Rating Scale
